# Post-Stroke Recovery: A Review of Hydrogel-Based Phytochemical Delivery Systems

**DOI:** 10.3390/gels11040260

**Published:** 2025-04-01

**Authors:** Irina Musa, Alexandra Daniela Rotaru-Zavaleanu, Veronica Sfredel, Madalina Aldea, Andrei Gresita, Daniela Gabriela Glavan

**Affiliations:** 1 Department of Psychiatry, University of Medicine and Pharmacy of Craiova, 200349 Craiova, Romania; irinamusa23@yahoo.com (I.M.); daniela.glavan@umfcv.ro (D.G.G.); 2 Doctoral School, University of Medicine and Pharmacy Craiova, 200349 Craiova, Romania; 3 Experimental Research Centre for Normal and Pathological Aging, University of Medicine and Pharmacy of Craiova, 200349 Craiova, Romania; alexandra.rotaru@umfcv.ro (A.D.R.-Z.); andrei.gresita@umfcv.ro (A.G.); 4 Department of Epidemiology, University of Medicine and Pharmacy of Craiova, 2–4 Petru Rares Str., 200349 Craiova, Romania; 5 Department of Physiology, University of Medicine and Pharmacy of Craiova, 2–4 Petru Rares Str., 200349 Craiova, Romania

**Keywords:** stroke rehabilitation, hydrogel delivery systems, phytochemicals, neuroprotection, neurogenesis

## Abstract

Stroke remains a leading cause of disability worldwide, underscoring the urgent need for novel and innovative therapeutic strategies to enhance neuroprotection, support regeneration, and improve functional recovery. Previous research has shown that phytochemicals such as curcumin, tannic acid, gallic acid, ginsenosides, resveratrol, and isorhamnetin display extensive neuroprotective properties, including antioxidant, anti-inflammatory, and anti-apoptotic effects. These natural compounds could also promote neurogenesis, angiogenesis, and the preservation of the blood–brain barrier. Despite their promising bioactivities, clinical application is often limited by poor solubility, bioavailability, and suboptimal pharmacokinetics. Hydrogels offer a promising solution by encapsulating and controlling the gradual release of these phytochemicals directly at the site of injury. Recent advancements in hydrogel formulations, constructed from biopolymers and functionalized using nanotechnological approaches, could significantly improve the solubility, stability, and targeted delivery of phytochemicals. Controlled release profiles from pH-sensitive and environment-responsive hydrogels could ensure that the compounds’ therapeutic effects are optimally timed with individual and critical stages of post-stroke repair. Moreover, hydrogel scaffolds with tailored material properties and biocompatibility can create a favorable microenvironment, reducing secondary inflammation, enhancing tissue regeneration, and potentially improving functional and cognitive outcomes following stroke. This review explores the potential of integrating phytochemicals within hydrogel-based delivery systems specifically designed for post-stroke recovery. The design and synthesis of biocompatible, biodegradable hydrogels functionalized especially with phytochemicals and their applications are also discussed. Lastly, we emphasize the need for additional robust and translatable preclinical studies.

## 1. Background

Stroke is the second leading cause of death and a significant contributor to disability globally, affecting individuals, caregivers, and the healthcare system [1]. Globally, stroke affects an estimated 15 million individuals annually [2], leading to approximately 5 million deaths and leaving another 5 million survivors permanently disabled [3]. This neurological emergency is characterized by a sudden disruption of blood flow to the brain caused by either a blockage in the cerebral vasculature due to small vessel arteriolosclerosis, cardioembolism, or large-artery atherothromboembolism (ischemic stroke), or the rupture of a blood vessel, most commonly caused by macrovascular lesions (vascular malformations, aneurysms, cavernomas) or venous sinus thrombosis (hemorrhagic stroke) [4]. In response to injury, glial cells proliferate, contributing to a chronic inflammatory environment that can last for weeks. Although initially beneficial, this inflammatory reaction and glial cell growth aggravate the damage of the neurovascular unit, worsening recovery outcomes [5]. Either ischemia or hemorrhage can cause extensive neuronal damage, resulting in debilitating deficits, including motor impairment (spasticity and weakness) [6], cognitive dysfunction (attention, memory, language, and orientation dysfunction) [7], and emotional disturbances such as post-stroke depression (PSD) [8].

Notably, PSD is the most common and severe neuropsychiatric complication following a cerebrovascular event, affecting about one-third of stroke survivors, with peak prevalence in the first year [9]. Over half of those affected experience persistent symptoms beyond three months, worsening recovery and quality of life [10]. PSD arises from neurochemical, structural, and systemic mechanisms [11] and presents with mood disturbances, cognitive deficits, psychomotor changes, sleep issues, and fatigue [12]. It increases mortality [13], suicidal ideation [14], and post-stroke impairments [15], ultimately limiting rehabilitation and long-term outcomes.

While long-term treatment of PSD involves both pharmacological and nonpharmacological approaches, in recent years, various phytochemicals have been tested for their potential therapeutic effect, wide availability, and low toxicity [16]. Moreover, modern technologies such as hydrogel-based delivery systems can enhance the therapeutic efficacy of phytochemicals by overcoming limitations such as poor solubility, low bioavailability, and rapid metabolism [17]. This targeted and controlled release approach could in turn provide sustained neuroprotection, promote neuroregeneration, and improve both motor and cognitive outcomes post-stroke, together with conventional treatment modalities. By integrating the latest advances in both phytochemical research and hydrogel technology for stroke recovery, this review aims to offer a comprehensive synthesis of preclinical evidence and mechanistic insights. Interestingly, this review also identifies critical gaps in clinical translation, underscoring the need for new, specialized preclinical setups.

A comprehensive literature search was conducted using multiple scientific databases, including PubMed, Scopus, and Web of Science. The keywords used for the search were “stroke”, “hydrogel delivery systems”, and “phytochemicals”. The search was limited to studies published in English from January 2004 to January 2025. Both in vivo and in vitro studies were included. A panel of two independent reviewers extracted data from the selected studies. Information was synthesized to emphasize the pharmacological properties of six of the most used phytochemicals, their bioavailability challenges, and the novel delivery systems employed, particularly hydrogels.

## 2. Current Stroke Treatment

Currently, the fundamental treatment for ischemic stroke is represented by the timely removal of the thrombus to restore cerebral blood flow and prevent irreparable neuronal damage [18]. Notably, the sole FDA-approved pharmaceutical treatment for IS is intravenous administration of tissue plasminogen activator (tPA) [19]. However, tPA’s restricted treatment window (<4.5 h) and accompanying bleeding risk render it relevant to less than 5% of patients [20]. Likewise, endovascular thrombectomy (ET) has a short therapeutic window (<6 h) and limited availability in specific sites. Both therapies involve risks of cerebrovascular consequences and hemorrhagic transformation [21,22]. Furthermore, effective revascularization may cause ischemia/reperfusion injury due to increased reactive oxygen species production [23]. Treatment approaches for hemorrhagic stroke differ, including surgical procedures such as hematoma evacuation, coiling, and aneurysm clipping intended to repair damaged vessels and lower intracranial pressure [24]. Antihypertensives, anticonvulsants, and therapies that decrease cerebral edema are crucial components of pharmacological therapy for managing various neurological conditions. For instance, antihypertensives such as beta-blockers (e.g., propranolol) and ACE inhibitors (e.g., lisinopril) are employed to manage blood pressure levels, which is vital in the context of stroke prevention and minimizing further brain injury. Anticonvulsants like levetiracetam and valproic acid are used to control seizures that can occur as a result of brain injuries or neurological disorders. Additionally, treatments aimed at reducing cerebral edema, such as osmotic diuretics (e.g., mannitol) and corticosteroids (e.g., dexamethasone), play a significant role in decreasing intracranial pressure and alleviating swelling in the brain tissue [25]. However, patients who have suffered a stroke typically require lifelong treatment to adequately manage their cognitive, motor and mood symptoms. Over time, however, the efficacy of such treatments may decrease, potentially necessitating adjustments or changes in therapeutic strategies. Additionally, long-term use of certain medications can lead to adverse effects due to toxicity. As such, continuous evaluation and adaptation of treatment plans are essential to address the evolving needs of stroke survivors [26]. This underscores the necessity for exploring alternative treatment approaches, such as the use of phytochemicals, which may offer fewer side effects and additional therapeutic benefits. Furthermore, the destruction of the blood–brain barrier in stroke patients complicates the delivery of these substances, necessitating the development of innovative drug delivery vehicles that can effectively target affected brain areas.

Promising new treatments also include gene therapy, cellular reprogramming, stem cell therapies, and exosome-based interventions [27]. For instance, preclinical studies have demonstrated the advantages of mesenchymal and neural stem cells through neuroprotection, angiogenesis, and immunomodulation [28], though issues like poor cell survival, tumorigenesis and long-term effects persist [29]. However, it is still unclear what the best cell type, dosage, delivery method, and timing are [30]. Furthermore, by turning resident glial cells into neurons, in vivo cellular reprogramming techniques that include transcription factors like NeuroD1, Ascl1, and Neurogenin2, and RNA-based gene editing, also show promise [31,32]. However, translating such studies into clinical settings is extremely challenging, as they often involve unknown long-term risks. The complexities of genetic and cellular treatments pose significant hurdles for implementation in clinical trials. First, the precise control over cellular behavior required for safety and efficacy is difficult to achieve. Additionally, the potential for off-target effects and genetic instability can lead to unintended consequences. Regulatory concerns also play a critical role, as the approval process for these novel therapies is stringent and often prolonged, reflecting the high standards needed to ensure patient safety [33]. This underscores the necessity for exploring alternative treatment approaches, such as the use of phytochemicals, which may offer fewer side effects and new therapeutic benefits. However, the stroke-induced disruption of the blood–brain barrier and the accompanying inflammatory response limit substance delivery, necessitating innovative drug delivery systems to precisely target affected brain regions. In a clinical setup, given the drawbacks of existing therapies, biomaterial-based interventions, particularly hydrogels, offer perhaps one of the most promising approaches. These materials could facilitate the transport, preservation, and enhancement of therapeutic agents [34]. Furthermore, utilizing natural phytochemicals, which carry minimal risk for toxicity, in combination with biocompatible gels made from collagen, alginate, or other biological compounds, could significantly increase the likelihood of receiving the necessary regulatory clearance. Moreover, such approaches are likely to elicit a more positive response from patients and caregivers who may be more willing to opt for these less invasive and more naturally derived treatment options [35]. This review aims to discuss these innovative treatment modalities and their potential in clinical practice.

## 3. Hydrogels in Stroke

### 3.1. Acute Applications

Hydrogels are hydrophilic, three-dimensional polymer networks with high water retention capacity, making them valuable in stroke therapy due to their biocompatibility, tunable mechanical properties, and extracellular matrix-mimicking characteristics [36]. To this end, their primary application in ischemic stroke is as drug delivery systems, enabling the controlled, sustained release of therapeutic agents within the ischemic penumbra [37]. This localized delivery could optimize drug bioavailability while minimizing systemic exposure, which is particularly crucial during the acute phase [38]. Furthermore, stimulus-responsive hydrogels can adjust drug release based on microenvironmental changes, such as pH or enzymatic activity, enhancing therapeutic efficacy [39].

**Figure 1:**
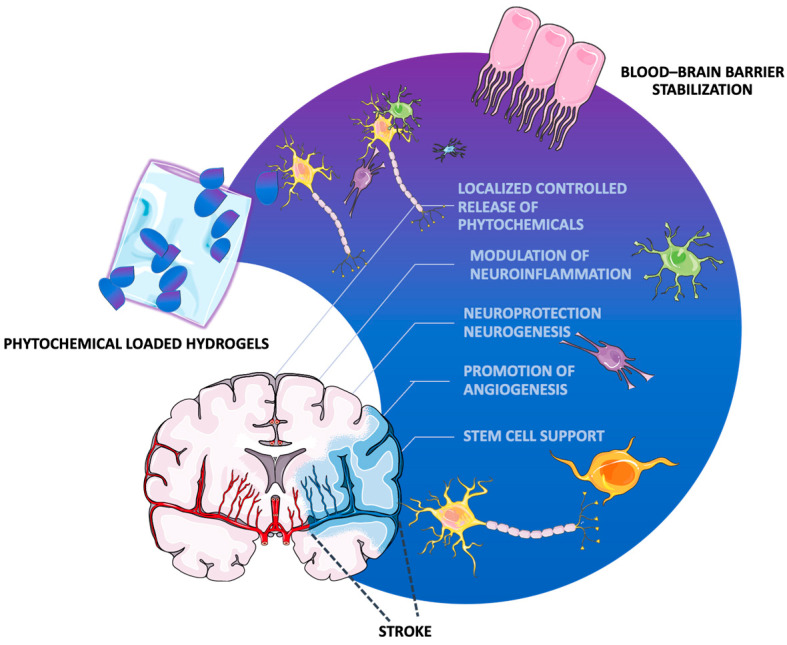
Mechanisms of action of phytochemical loaded hydrogels in stroke recovery. The selected artwork was taken or adapted from pictures provided by Servier Medical Art (Servier; https://smart.servier.com/, accessed on 13 March 2025), licensed under a Creative Commons Attribution 4.0 Unported License [45].

Beyond drug delivery, hydrogels facilitate neuroprotection and post-stroke tissue repair by providing a biocompatible three-dimensional microenvironment that supports stem cell survival, differentiation, and therapeutic efficacy in stroke recovery [40]. Moreover, they facilitate neuroprotection, angiogenesis, and synaptic plasticity through the sustained delivery of bioactive molecules such as BDNF and VEGF [41]. Additionally, hydrogels can modulate apoptotic pathways, enhance tissue regeneration, and provide controlled degradation rates, ensuring long-term therapeutic effects crucial for post-stroke recovery and functional restoration [35]. Hydrogels might also mitigate secondary ischemic injury, particularly reperfusion-associated oxidative stress and inflammatory damage, by modulating the inflammatory response and scavenging reactive oxygen species, helping preserve neuronal integrity [42]. Moreover, hydrogels could contribute to blood–brain barrier stabilization, either by delivering reparative agents (growth factors, peptides, stem cells) or by serving as a physical barrier to reduce cerebral edema and hemorrhagic transformation [43] (Figure 1). Although still in early research, the application of nanotechnologies, including hydrogels, in hemorrhagic stroke could offer significant therapeutic potential by delivering hemostatic and neuroprotective agents directly to the hemorrhagic site. Notably, such gels could hold the ability to conform to irregular lesion cavities, reducing bleeding and fostering tissue repair [44].

Notably, besides their documented biocompatibility and bioavailability, the biodegradability and long-term safety of hydrogels are essential considerations. The degradation of hydrogels depends on their composition, crosslinking density, and environmental conditions, with mechanisms including solubilization, ionization resulting in dissolution, chemical hydrolysis, and enzymatic degradation [46]. Natural polymers, such as hyaluronic acid, collagen, and alginate, exhibit favorable biocompatibility and enzymatic degradation, minimizing toxic byproduct accumulation [47]. However, their long-term stability and mechanical performance must be optimized to ensure sustained functionality without compromising hydrogel integrity over time [48]. Another fundamental concern is related to the predictability and biocompatibility over extended periods, preserving mechanical integrity [49] and avoiding the accumulation of harmful degradation byproducts that could adversely affect tissue health [50].

### 3.2. Long-Term Applications

Progressing from acute stroke management, long-term stroke rehabilitation also remains a major challenge, with motor impairment being a key focus of recovery effort [51]. Hydrogel therapy represents a promising strategy for enhancing motor recovery following stroke by providing a localized, controlled-release system for neuro-regenerative agents. For instance, exosome-loaded hydrogels, such as adhesive hyaluronic acid hydrogels, have demonstrated significant improvements in motor function by promoting cerebral angiogenesis and reducing inflammation, as evidenced by increased neurovascular remodeling and behavioral recovery in ischemic stroke models [52]. Similarly, the incorporation of brain-derived neurotrophic factor within hydrogels has been shown to facilitate motor system recovery by inducing axonal sprouting and enhancing neuronal migration into peri-infarct regions, leading to functional improvements in both rodent and non-human primate models [53]. The combination of hydrogels with extracellular vesicles from mesenchymal stem cells could also provide structural support while optimizing the retention and distribution of EVs, thereby enhancing motor recovery without the risks of direct stem cell transplantation [54].

Another application of hydrogels in long-term post-stroke recovery is the management of post-stroke depression. Their ability to provide targeted, sustained delivery of antidepressants and neurotrophic factors supports neuronal survival, synaptic plasticity, and tissue repair [55,56]. Moreover, hydrogels could potentially co-deliver agents addressing neuroinflammation and neurotransmitter deficits, promoting neural repair while mitigating mood disorders [57]. The co-delivery system not only ensures the simultaneous arrival of these agents at the injury site but also maintains their relative concentrations at optimal therapeutic levels. However, it is worth mentioning that despite their therapeutic potential, hydrogel-based therapies face challenges in clinical translation due to immunogenic risks, difficulties in controlling drug release and degradation rates, and scalability issues that affect uniformity, reproducibility, and regulatory approval [58]. Furthermore, the production of hydrogels requires specialized equipment and personalized formulations, suggesting that mass production may not be feasible. While hydrogels provide localized methods of delivery, it is important to recognize that in the context of stroke, the entire brain may be affected, thus necessitating generalized treatment approaches as well [59]. Additionally, the efficacy of hydrogel applications can be significantly influenced by patient-specific factors such as comorbidities and age. In stroke management, where patients are predominantly elderly, the natural healing processes are often impaired, which could lead to reduced effectiveness of hydrogels in promoting recovery [60]. These factors collectively highlight the complexity of developing and implementing hydrogel-based interventions in a clinical setting.

## 4. Phytotherapy in Stroke

**Figure 2:**
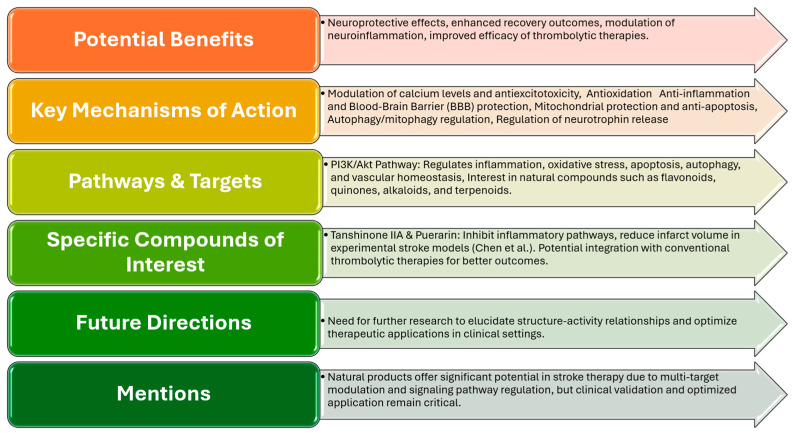
Phytotherapy in stroke recovery [64].

Phytotherapy has gained attention in the context of stroke rehabilitation due to its potential neuroprotective effects and ability to enhance recovery outcomes. Various phytochemicals have been identified for their neuroprotective properties, particularly in the context of ischemic stroke. For example, Xu and colleagues [61] provide a comprehensive review of 148 phytochemicals that exhibit neuroprotective activities in experimental models of ischemic stroke, highlighting their role in mitigating neuronal damage and promoting recovery via various strategies, including modulation of calcium levels and anti-excitotoxicity, antioxidation, anti-inflammation and BBB protection, mitochondrial protection and antiapoptosis, autophagy/mitophagy regulation, and control of neurotrophin release. In addition to their neuroprotective effects, phytochemicals may also play a role in modulating neuroinflammation, which is a significant contributor to stroke pathology. Hung et al. emphasize the health-promoting effects of dietary phytochemicals in targeting the NLRP3 inflammasome, a key player in neuroinflammation associated with neurological disorders, further supporting recovery and improving long-term outcomes for stroke survivors [62]. Furthermore, Liu et al. reviewed two decades of research on anti-ischemic stroke drugs targeting the PI3K/Akt pathway, highlighting its role in regulating inflammation, oxidative stress, apoptosis, autophagy, and vascular homeostasis, and emphasizing the growing interest in natural compounds—such as flavonoids, quinones, alkaloids, and terpenoids—in stroke therapy [63] (Figure 2).

Moreover, specific natural compounds have shown promise in enhancing the efficacy of thrombolytic therapies. Chen et al. discuss the potential of Tanshinone IIA and Puerarin, which inhibit inflammatory pathways and reduce infarct volume in experimental stroke models, suggesting that integrating these phytochemicals with conventional treatments could improve outcomes for patients experiencing acute ischemic strokes [64]. Overall, natural products hold significant potential as neuroprotective agents in stroke therapy due to their ability to modulate multiple targets and signaling pathways, exerting both direct and indirect effects on key regulatory enzymes and proteins. However, further research is needed to elucidate their structure–activity relationships and optimize their therapeutic application in clinical settings [65].

## 5. Hydrogel-Based Phytochemical Delivery Systems

Phytochemical-loaded hydrogels have garnered significant attention in various medical applications, primarily due to their ability to incorporate bioactive plant compounds that enhance therapeutic efficacy. Among the most notable applications are wound healing, cancer treatment, and neurological recovery, particularly in stroke rehabilitation [34]. One of the most prominent applications of phytotherapeutic hydrogels is in wound healing. These hydrogels can be formulated to include essential oils and other phytochemicals known for their antibacterial, anti-inflammatory, and antioxidant properties. For instance, essential oils such as clove and peppermint have been integrated into hydrogel matrices to promote wound healing by accelerating collagen formation and reducing oxidative stress, as well as supporting the proliferation of human dermal fibroblasts, indicating their potential as advanced wound dressings [66]. Moreover, they have demonstrated potential in the treatment of diabetic ulcer lesions [67], second-degree burns [68], and chronic cutaneous lesions [69]. Another important application is in cancer treatment, as many patients with cancer utilize phytotherapeutic products alongside conventional treatments, seeking to mitigate side effects and enhance therapeutic outcomes [70]. For example, bitter melon extract has been investigated for its potential in breast and gynecological cancer prevention, showcasing the efficacy of phytochemicals in cancer therapy [71]. Phytotherapeutic hydrogels have demonstrated potential in cancer therapy by integrating plant-derived compounds with hydrogel matrices to enhance bioavailability, targeted delivery, and sustained release of anticancer agents. Studies on coumarin and p-coumaric acid-loaded fibrin hydrogels have shown their ability to induce apoptosis and autophagy in colorectal cancer cells via the PI3K/Akt/mTOR and AMPK/mTOR pathways, highlighting their role in tumor suppression and potential applications in oncological treatments [72].

In the field of neurological recovery, particularly following stroke, phytotherapeutic hydrogels are starting to be explored for their potential to enhance cognitive function and promote neural regeneration. For example, direct self-assembly of the natural product into a stable, nanofiber hydrogel enables sustained drug release and efficient cellular uptake, thereby effectively inhibiting NFκB signaling and attenuating neuroinflammation with minimal cytotoxicity. This phytotherapeutic hydrogel approach holds significant promise for developing innovative, low-toxicity therapies to combat neuroinflammatory disorders [73]. Notably, several phytotherapeutic compounds have demonstrated potential in stroke management, with some investigated as phytotherapeutic hydrogels in stroke therapy and others explored in different medical contexts. This paper will examine six such compounds (curcumin, tannic acid, gallic acid, ginsenosides, resveratrol, and isorhamnetin), evaluating their potential as phytotherapeutic hydrogels for stroke treatment and post-stroke recovery.

### 5.1. Curcumin in Hydrogel-Based Delivery Systems

Curcumin, a polyphenolic compound derived from turmeric, has been demonstrated to directly prevent cerebral ischemia through a number of methods, including promoting neurogenesis [74], preventing endoplasmic reticulum stress [75], and blocking mitochondrial apoptosis [76]. It has also been revealed that curcumin exhibits neuroprotective effects through shifting microglia/macrophage polarization from a proinflammatory phenotype to an anti-inflammatory state [77], while also attenuating microglial pyroptosis, enhancing white-matter integrity, and improving functional outcomes, through NF-κB signaling suppression and subsequent NLRP3 inflammasome inhibition [78].

In the context of post-stroke depression, curcumin demonstrates antidepressant-like properties through multiple pathways. Firstly, it modulates neurotransmitter levels, including dopamine, norepinephrine, and serotonin, and inhibits the expression of monoamine oxidase enzymes. This helps in reducing the inflammatory response by regulating the production of pro-inflammatory markers. Additionally, curcumin aids in repairing neurodegeneration and enhances neurogenesis and neuronal plasticity, which is evidenced by increased levels of brain-derived neurotrophic factor (BDNF). Further, curcumin improves the activities of antioxidant enzymes, decreases nitric oxide levels, and regulates mitochondrial disturbances. It also moderates hypothalamus–pituitary–adrenal (HPA) disturbances, contributing to its overall therapeutic effects in managing post-stroke depression [79]. Recent studies have demonstrated that curcumin-loaded hydrogels can effectively modulate the inflammatory response following stroke. For instance, Zhang et al. reported that a curcumin-loaded hydrogel with dual reactive oxygen species (ROS)-scavenging capabilities can regulate microglial polarization, promoting rehabilitation after stroke [80]. This is especially important, as the inflammatory response following a stroke is greatly influenced by microglial activation, and regulating this response can have a big impact on recovery results. Moreover, the physicochemical properties of the hydrogels can be tailored to optimize curcumin delivery. For example, Yang et al. highlighted the fabrication of pH-sensitive biopolymer hydrogels that exhibited distinct release profiles for curcumin, indicating that the hydrogel composition can be adjusted to achieve desired release kinetics [81]. Such modifications are crucial for ensuring that curcumin is released in a manner that aligns with the physiological conditions present in the post-stroke environment.

The sustained release of curcumin from hydrogels also addresses the challenge of its poor water solubility, which limits its therapeutic application [82]. Chen et al. emphasized the importance of using nanocarrier-assisted delivery systems to enhance curcumin’s solubility and bioactivity, particularly when integrated into hydrogels designed for spinal cord injury repair [83]. This approach not only improves the bioavailability of curcumin but also enhances its therapeutic efficacy in modulating the post-stroke microenvironment.

In addition to the mechanical and release properties of the hydrogels, their biocompatibility is paramount. Madech et al. demonstrated that curcumin encapsulated in chitosan-based hydrogels exhibited non-cytotoxic effects on normal cells, suggesting that these systems can be safely used in vivo [84]. A novel EDV/Cur/NapFFY hydrogel, co-assembling curcumin and edaravone with a peptide hydrogelator, has been shown to improve drug bioavailability and facilitate targeted delivery to ischemic sites. In vitro studies demonstrate that this hydrogel enables sustained release of curcumin and edaravone for up to two weeks, while in vivo experiments indicate its ability to promote brain plasticity and enhance functional recovery in a photothrombotic mouse model, underscoring the therapeutic potential of peptide-based hydrogels in optimizing neuroprotection and repair following ischemic stroke [85] (Table 1).

### 5.2. Tannic Acid in Hydrogel-Based Delivery Systems

Tannic acid (TA) is a naturally occurring polyphenol that has garnered increasing interest due to its beneficial pharmacological effects, which include anti-inflammatory, antioxidative, and antibacterial effects [86], as well as its versatility for the creation of multifunctional hydrogels with adhesive, stretchable, self-healing, and biodegradable qualities [87]. Tannic acid has been shown to enhance cell proliferation and exhibit strong antioxidant activity, which is crucial in mitigating oxidative stress following a stroke [88]. The incorporation of tannic acid into hydrogel matrices could potentially improve therapeutic outcomes by providing a conducive environment for cellular repair and regeneration [89]. Tannic acid also exhibits neuroprotective and antidepressant-like effects in depression models by modulating key neurochemical pathways. In an LPS-induced mouse model of depression, TA administration for seven days reduced acetylcholinesterase activity in both healthy brain tissue and LPS-treated mice, suggesting enhanced cholinergic function. Additionally, TA prevented LPS-induced dysregulation of ion pumps, restoring Na^+^/K^+^-ATPase activity in the cerebral cortex, hippocampus, and striatum and normalizing Ca^2+^-ATPase activity, which was increased in the cortex and decreased in the hippocampus following LPS exposure [90].

Yu and colleagues [91] have shown that tannic acid may trap free radicals, which is advantageous in preventing oxidative destruction of biomolecules. Research suggests that hydrogels containing tannic acid can efficiently scavenge free radicals, thereby minimizing oxidative damage in post-stroke circumstances. Furthermore, the antioxidant capacity of tannic acid is enhanced when it is embedded in hydrogels, allowing for sustained release and prolonged activity in the affected tissues [92]. The biocompatibility of tannic acid-based hydrogels is another critical aspect that supports their application in post-stroke therapy. Kaczmarek and colleagues [88] reported that hydrogels made from tannic acid and sodium alginate exhibited favorable biocompatibility with fibroblast cells, indicating their potential for use in biomedical applications. Additionally, tannic acid’s ability to form stable complexes with metal ions can be leveraged to enhance the mechanical properties of hydrogels, making them suitable for tissue engineering and drug delivery applications [93]. This is particularly relevant for nerve recovery, where the mechanical integrity of the hydrogel can support repair processes.

Moreover, the anti-inflammatory properties of tannic acid can play a significant role in post-stroke recovery. Tannic acid has been shown to suppress nitric oxide production in stimulated macrophages, which is a critical mediator of inflammation [92]. By incorporating tannic acid into hydrogels, it may be possible to create a localized anti-inflammatory environment that promotes healing and reduces secondary injury following a stroke. After post-treating the enzymatically crosslinked chitosan-alginate hydrogels with TA, Jafari et al. discovered that the hydrogel’s adhesiveness, antioxidant, and antibacterial qualities, as well as its cytocompatibility and cell proliferation, were all markedly enhanced and better suited for biomedical applications [94]. In the context of post-stroke recovery, Liu and Zhang et al. [95] proposed that tannic acid hydrogel, formed with carboxymethyl chitosan, effectively modulates microglial polarization and enhances neuroplasticity. Their findings demonstrate that TA gel sustainably releases TA, shifting microglia towards an anti-inflammatory phenotype by downregulating CD16 and IL-1β while upregulating CD206 and TGF-β in oxygen and glucose-deprived (OGD) BV2 cells, promoting synaptic repair in OGD N2a cells in vitro. In stroke mice, TA gel injection reduced CD16/iNOS expression, increased CD206 expression, and enhanced neuroplasticity, as evidenced by PSD95/Vglut1 colocalization and Golgi staining. Additionally, motor function recovery was significantly improved, and western blot analysis confirmed that TA gel regulates microglial polarization via the NF-κB pathway, suggesting that TA gel may serve as a viable injectable therapy for post-stroke rehabilitation [95] (Table 2).

### 5.3. Gallic Acid in Hydrogel-Based Delivery Systems

Gallic acid (3,4,5-trihydroxybenzoic acid) is a phenolic acid widely distributed in many different families of higher plants, which can be found in different concentrations in common foodstuffs such as blueberries, blackberries, strawberries, plums, grapes, mango, cashew nuts, hazelnuts, walnuts, tea, and wine [96]. Gallic acid has demonstrated extensive neurobiological effects across various neurological and psychiatric disorders, including ischemic and hemorrhagic stroke, Alzheimer’s disease, Parkinson’s disease, depression (including post-stroke depression), anxiety, psychosis, neuroinflammation, neuropathic pain, brain tumors, and sedation [97]. Gallic acid further demonstrated cerebroprotective effects against cerebral ischemia/reperfusion (I/R) injury in rats by improving behavioral impairments, including gait, sensorimotor function, and memory, and enhancing antioxidant defenses, highlighting its potential as a therapeutic agent for brain injury recovery [98]. It has also been shown to mitigate cerebral ischemia/reperfusion injury by modulating mitochondrial dysfunction through preserving mitochondrial membrane potential, reducing intracellular and mitochondrial reactive oxygen species (ROS), inhibiting mitochondrial permeability transition pore (MPTP) opening, and enhancing ATP production. In vivo, GA decreased infarct size, reduced neuronal apoptosis through inhibition of cytochrome C release, and attenuated hypoxia/reoxygenation-induced damage, demonstrating its potential as a protective agent in brain ischemia [99]. Moreover, GA also exerts neuroprotective effects in cerebral ischemia by preserving blood–brain barrier integrity, reducing brain edema, and improving neurological outcomes, with its administration in a middle cerebral artery occlusion (MCAO) model mitigating microglial activation, potentially by inhibiting M1 polarization, thereby contributing to reduced ischemia/reperfusion-induced injury and enhanced neuronal survival [100]. In the context of post-stroke depression (PSD), gallic acid and its analogues (M3OMG, P3OMG) exhibited antidepressive-like effects by reducing oxidative stress. In a mouse model of PSD, their administration (25–50 mg/kg) improved depressive behaviors, enhanced antioxidant enzyme activity (SOD, Cat), decreased lipid peroxidation (TBARS), and restored glutathione (GSH) levels [101].

Despite its many health-associated benefits, its utility is limited due to poor absorption, low bioavailability, high metabolism, and high clearance rate [102]. To overcome these drawbacks, formulation development and nanotechnological strategies are employed that incorporate GA, thereby enhancing its pharmacological efficacy. These strategies include solid-lipid nanoparticles, liposomes, emulsions, phospholipid complexes, niosomes, solid-lipid microparticles, microemulsions, micelles, polymeric nanoparticles, lipid–polymer hybrids, conjugates, Ag nanoparticles, silica nanoparticles, cyclodextrin complexes, and hydrogels [103]. One study demonstrated the neuroprotective effects of gallic acid against cerebral ischemia/reperfusion injury (CIRI) through the development of GA-loaded o-carboxymethyl chitosan nanoparticles (GA-NPs) that have enhanced GA’s bioavailability, prolonged its half-life, and improved its therapeutic efficacy in ischemic stroke models by reducing oxidative stress and inflammation [104]. In regard to hydrogels, one review article highlighted the potential of gallic acid as a key component in multifunctional hydrogel systems due to its strong adhesion, antioxidant, and antibacterial properties, serving as both a hydrogel crosslinker and a bioactive additive, offering advantages over catechol-based hydrogels and greater chemical modification flexibility than polymeric tannic acid [105].

Although yet to be researched, gallic acid-based hydrogels hold significant potential for post-stroke recovery by addressing critical pathological features such as oxidative stress, neuroinflammation, and blood–brain barrier dysfunction. In one study, a gallic acid-based hydrogel encapsulating stem cell-derived exosomes demonstrated robust antioxidant activity and sustained release properties, effectively reducing ROS, shifting microglia to the M2 anti-inflammatory phenotype, and promoting neuroregeneration in traumatic brain injury (TBI) models [106]. Another study reported similar success with an injectable gallic acid-grafted hyaluronic acid (HGA) hydrogel, which reduced oxidative stress via the Nrf2/HO-1 pathway, lowered levels of proinflammatory cytokines like TNF-α and IL-6, and enhanced neurogenesis and motor recovery [107]. These findings highlight the potential of gallic acid hydrogels in managing stroke recovery, given the parallels between TBI and stroke pathologies.

The regenerative potential of gallic acid-functionalized hydrogels extends beyond neurological applications. In large bone defects, gallic acid-grafted gelatin (GGA) hydrogels combined with zinc-strontium phosphate (ZSP) enhanced the bone microenvironment by promoting angiogenesis, scavenging ROS, and inducing osteogenesis [108]. These properties, especially the promotion of vascular regeneration, suggest potential applicability to ischemic brain conditions like stroke. Similarly, polyvinyl alcohol–gallic acid (PVA–GA)-based hydrogels developed for burn wound healing demonstrated multifunctionality, including self-healing, antibacterial, antioxidant, and proangiogenic activities. These hydrogels accelerated tissue repair, reduced inflammation, and exhibited pH-responsive drug release, indicating their suitability for managing complex repair environments, as well as highlighting their potential in post-stroke recovery [109] (Table 3).

### 5.4. Ginsenosides in Hydrogel-Based Delivery Systems

Ginseng, the root of Panax ginseng, has been widely used to treat cerebrovascular diseases in Asian countries. Ginsenosides are the major bioactive components of ginseng, responsible for its pharmacological activities [110]. Ginsenosides have been extensively studied for their therapeutic potential across various medical fields, including cardiovascular health [111], diabetes management [112], cancer treatment [113], and neuroprotection. The neuroprotective effects of both major ginsenosides and minor ginsenosides against cerebral ischemia are mediated by the regulation of excitotoxicity, Ca^2+^ overload, inflammation, mitochondria dysfunction, oxidative stress, apoptosis, pyroptosis, autophagy, BBB permeability, angiogenesis, and neurogenesis [114]. Among individual ginsenosides, Ginsenoside Rg1 (G-Rg1) has been shown to significantly improve neurological function and reduce infarct volume in experimental stroke models, exerting its neuroprotective effects by enhancing neurogenesis, preserving BBB integrity, and exhibiting antioxidant and anti-inflammatory properties [115]. G-Rg1 has also been reported to promote angiogenesis and improve neurological outcomes in ischemic stroke models by activating vascular endothelial growth factor (VEGF) pathways [116]. Ginsenoside Rb1 (G-Rb1) plays a crucial role in reducing brain edema, improving cerebral circulation, and promoting neurogenesis, and also exhibiting potent anti-apoptotic and anti-oxidative stress effects, which contribute to its neuroprotection in ischemic stroke [117]. Ginsenoside Rd (G-Rd) has been found to attenuate infarct volume and improve neurological recovery after ischemia/reperfusion injury, protect against oxidative stress, modulate calcium homeostasis, and inhibit inflammatory pathways, making it a promising neuroprotective agent [118]. Collectively, these ginsenosides represent potential candidates for stroke therapy, warranting further clinical investigation.

A meta-analysis of 23 preclinical studies highlighted the significant antidepressant-like effects of ginsenosides, particularly Rg1, which showed notable behavioral improvements in forced swimming and sucrose preference tests [119]. Given their neuroprotective properties, ginsenosides, especially Rg1, may be promising for post-stroke recovery and post-stroke depression by supporting neuronal repair and alleviating mood disturbances [119]. Despite their benefits, ginsenosides exhibit low oral bioavailability due to their poor stability in the gastrointestinal tract, limited membrane permeability, and extensive intestinal and hepatic first-pass metabolism, with their primary gastrointestinal metabolism involving deglycosylation by intestinal microflora, further influencing their pharmacokinetics [120]. To enhance bioavailability and therapeutic efficacy, advanced drug delivery systems—including hydrogels, liposomes, ethosomes, transfersomes, metal/metal oxide systems, micro/nanoemulsions, polymeric micro/nanoparticles (NPs), liposomes, transfersomes, and micelles—have been developed to improve stability, facilitate controlled release, and enable targeted delivery [121].

The application of ginsenosides in hydrogels offers significant potential for advancing post-stroke recovery by leveraging their proven therapeutic effects across various medical contexts. Research has shown that hydrogels encapsulating ginsenosides, such as Rb1 and Rg1, can enhance tissue repair and regeneration through multiple mechanisms. For instance, Rb1-loaded hydrogels demonstrated dose-dependent chondroprotective effects in osteoarthritis models by downregulating matrix metalloproteinases (MMPs), apoptotic markers like TNF-α and caspase-3, and oxidative stress via the suppression of the NF-κB, PI3K/Akt, and MAPK pathways [122]. Additionally, Rg1 has been found to improve liver injury by activating the Nrf2 signaling pathway and enhancing glutathione synthesis, further showcasing its anti-inflammatory and antioxidative properties [123]. In wound-healing studies, hydrogels loaded with ginsenosides such as Rg1 [124] and Rg3 [125] have shown exceptional biocompatibility and the ability to promote angiogenesis, collagen deposition, and tissue regeneration. This aligns with the neuroprotective and regenerative needs in post-stroke therapy, where angiogenesis and reduced inflammation are vital for recovery. Moreover, myocardial infarction studies reveal that Rb1-loaded hydrogels improve mitochondrial function, reduce fibrosis, and support angiogenesis, leading to significant restoration of cardiac function [126]. These outcomes suggest a similar potential for promoting neural and vascular repair in stroke recovery.

The integration of ginsenoside hydrogels into post-stroke care is further supported by their ability to provide localized, sustained delivery of bioactive compounds, addressing challenges such as oxidative damage, apoptosis, and neuroinflammation. Drawing from studies where hydrogels have successfully modulated immune responses and created bioactive environments for healing, such as cancer therapy with Rg3-loaded hydrogels, there is strong evidence to suggest their applicability in the complex post-stroke neuroinflammatory landscape [127]. While direct research on their use in stroke recovery is limited, the extensive data from other fields highlight their immense potential to revolutionize neurorehabilitation through targeted, sustained, and multifunctional therapeutic strategies (Table 4).

### 5.5. Resveratrol in Hydrogel-Based Delivery Systems

Resveratrol (3,5,4′-trihydroxystilbene) is a naturally occurring phytosterol that resembles estrogen and is mostly present in grapes, peanuts, blueberries, red wine, and other dietary components [128]. It pertains to the stilbene class that occurs in two isomers: cis and trans. Stilbenes, a type of secondary metabolite, eliminate free radicals and protect against chronic illnesses like diabetes, cancer, heart disease, and arteriosclerosis, exhibiting antioxidant, anti-aging, and anti-angiogenics proprieties [129]. Resveratrol exhibits neuroprotective properties by modulating synergistic pathways that regulate oxidative stress, inflammation, and cell death, offering therapeutic potential in stroke, as well as traumatic brain injury and spinal cord injury [130]. In a post-stroke context, it modulates microglial signaling pathways (AMPK, SIRT1, SOCS1), promotes M2 polarization, enhances astrocytic energy support by activating AMPK and inhibiting GSK-3β, reduces oxidative stress, and improves oligodendrocyte survival, collectively supporting post-stroke brain homeostasis [131]. Resveratrol also offers neuroprotection in ischemic stroke by activating SIRT1 and Nrf2 pathways to preserve blood–brain barrier integrity, upregulating antioxidant genes like SOD2 and NQO-1, and enhancing anti-apoptotic factors such as Bcl-2. Moreover, it decreases cerebral edema by downregulating aquaporin-4 and improves cerebral blood flow through nitric oxide production and reduced angiotensin II and endothelin-1, making resveratrol a promising treatment for mitigating stroke damage [132]. Resveratrol also shows promise in treating depression, including post-stroke depression, by regulating the HPA axis, reducing inflammation, and enhancing BDNF and neurogenesis. Effective in animal models at doses of 10–80 mg/kg/day, it may offer a natural alternative with fewer side effects, but further research is needed to validate its efficacy in humans [133].

As with previous molecular agents, resveratrol’s therapeutic potential is often limited by its poor solubility and low bioavailability, which significantly reduce its effectiveness in clinical applications. Its rapid metabolism and short half-life result in limited systemic circulation and reduced concentrations at target sites, hindering its full pharmacological potential [134]. Although resveratrol hydrogels have yet to be researched in post-stroke recovery, they hold significant promise by addressing key pathological mechanisms such as oxidative stress, inflammation, and impaired neurogenesis. Various studies have shown that hydrogels enhance the bioavailability and stability of resveratrol in other applications, allowing for its sustained release and targeted delivery. For example, nanostructured hydrogels improve drug permeability and therapeutic efficacy, as demonstrated in Alzheimer’s models where resveratrol enhanced cognitive function [135]. Another study on resveratrol-loaded chitosan (CS) nanoparticles in hyaluronic acid (HA) hydrogels for treating atopic dermatitis could be applied to post-stroke recovery, with the hydrogel’s ability to release resveratrol steadily and reduce oxidative stress and inflammation in skin cells suggesting it could also help mitigate neuronal damage and inflammation in stroke recovery [136]. In diabetic wound models, resveratrol-loaded hydrogels reduced pro-inflammatory cytokines, promoted angiogenesis, and accelerated healing through sustained release and modulation of the inflammatory microenvironment [137]. Additionally, composite hydrogels incorporating resveratrol and other agents have been effective in creating conducive environments for tissue regeneration, such as osteoporotic bone defects and chronic wounds [138]. These findings suggest that resveratrol hydrogels could be adapted for post-stroke therapy to promote angiogenesis, regulate inflammation, and support neural repair, offering a promising avenue for improving recovery outcomes (Table 5).

### 5.6. Isorhamnetin in Hydrogel-Based Delivery Systems

Isorhamnetin is one of the most important active ingredients in the fruits of *Hippophae rhamnoides* L. and the leaves of *Ginkgo biloba* L., possessing extensive pharmacological activities in cardiovascular and cerebrovascular protection and exhibiting anti-tumor, anti-inflammatory, and anti-oxidation proprieties [139]. For instance, in an ischemic stroke model, isorhamnetin reduced infarct volume, inhibited caspase-3 activity, and preserved blood–brain barrier integrity by reducing cerebral edema and upregulating tight junction proteins [140]. Moreover, by strengthening antioxidant defenses, decreasing cholinesterase activity, and raising BDNF levels in a scopolamine-induced model, Ishola et al. demonstrated that isorhamnetin enhances cognitive function, suggesting its significance in reducing oxidative stress and fostering synaptic plasticity [141]. Ekici et al. also demonstrated that isorhamnetin and kaempferol reduce oxidative stress and inflammation (via lower TNF-α, IL-1β, and IL-6) and restore BDNF levels in the prefrontal cortex and hippocampus, thereby alleviating LPS-induced anxiety and depression [142]. With the former concentrating on cognitive deficits and the latter on anxiety and depression, both studies outline the neuroprotective benefits of isorhamnetin, which are fueled by its modulation of the oxidative stress–inflammation–BDNF axis [141,142]. Additionally, in a lipopolysaccharide (LPS)-induced depression model, Gammoh et al. showed that isorhamnetin markedly improved the antidepressant efficacy of escitalopram, as indicated by a decrease in depressive-like behaviors in behavioral tests. Furthermore, in the prefrontal cortex and hippocampus, isorhamnetin restored decreased levels of Nrf2, BDNF, and HO-1, demonstrating its potential to supplement traditional antidepressant therapy through anti-inflammatory and antioxidant mechanisms [143].

Studies on cellular cultures also suggest that isorhamnetin holds promise for post-stroke recovery and the management of post-stroke depression through its neuroprotective, anti-inflammatory, and antioxidative properties. By reducing oxidative stress, inflammation, and apoptosis in human brain microvascular endothelial cells subjected to methylglyoxal (MGO) and oxygen–glucose deprivation (OGD), Li et al. showed that isorhamnetin prevents ischemia-induced cerebral vascular degeneration, particularly through the inhibition of Fas/FasL signaling and NF-κB nuclear translocation [144]. It alleviates ischemia/reperfusion-induced neuronal injury in a high-glucose environment by activating the Akt/SIRT1/Nrf2/HO-1 pathway in HT22 hippocampal neurons, indicating a potential role in mitigating oxidative stress and neuronal apoptosis following stroke [145]. Its suppression of neuroinflammation via inhibition of the NF-κB and Toll-like receptor 4 signaling pathways in BV2 microglia suggests relevance in reducing post-stroke neuroinflammatory responses, which are closely linked to depressive symptoms [146]. Additionally, isorhamnetin protects against rotenone-induced neurotoxicity in pc12 cells through modulation of the PI3K/Akt/GSK-3β/CREB pathway, which may contribute to neuronal survival and synaptic plasticity in stroke-affected regions [147]. Furthermore, its ability to enhance nerve growth factor-induced neurite outgrowth in pc12 cells points to a potential role in promoting neuronal repair and plasticity, essential processes for post-stroke recovery and resilience against depression [148].

Despite the promising biological activities of isorhamnetin, its bioaccessibility is significantly reduced during the intestinal digestion phase, necessitating strategies to enhance its stability and absorption capabilities. Efforts to improve the bioaccessibility and permeability of isorhamnetin glycosides have focused on understanding the effects of glycosylation and developing controlled delivery systems to maximize their therapeutic potential [149]. While specific studies on isorhamnetin and hydrogels are scarce, the existing literature on the beneficial effects of isorhamnetin suggests that it could be effectively incorporated into hydrogel systems for enhanced delivery and efficacy. Recent advancements in hydrogel-modified chromatography techniques have enabled the efficient screening and purification of active ingredients, including isorhamnetin, from herbal medicines. This approach can facilitate the development of targeted therapies that combine isorhamnetin with hydrogels, optimizing its delivery and enhancing its therapeutic effects in stroke recovery [150].

Hydrogel-based delivery systems have been shown to enhance the bioavailability and therapeutic efficacy of flavonoids, including isorhamnetin. Studies on 3-O-methylquercetin (3OMQ) incorporated into hydroxypropyl methylcellulose (HPMC) hydrogels with cyclodextrins demonstrated improved skin permeation and retention, suggesting a promising strategy for isorhamnetin formulations as well [151,152]. Additionally, gelatin-based nanofibers loaded with isorhamnetin glycosides (IRGs) have shown controlled release and biocompatibility with human fibroblast cells, further supporting the potential of polymer-based systems for isorhamnetin delivery [153]. Beyond polymeric approaches, biogenic silica carriers have been explored for encapsulating isorhamnetin using a novel microfluidic method, achieving significant encapsulation efficiency and controlled release [154]. Furthermore, 3D hydrogel scaffolds have demonstrated the neuroprotective effects of flavonoids in neural co-culture models, reinforcing the potential of isorhamnetin-loaded hydrogels in neurological applications [155]. Additionally, the synergistic effects of isorhamnetin with other neuroprotective agents or rehabilitation strategies could further enhance recovery outcomes. For example, combining isorhamnetin with brain-derived neurotrophic factor in hydrogel formulations could promote neurogenesis and functional recovery after stroke, potentially leveraging the unique properties of both compounds to maximize therapeutic benefits [53] (Table 6).

#### Isorhamnetin Delivery Strategies for Stroke Recovery

Isorhamnetin, together with the previously described phytochemicals, can in turn be administered through various routes, each with distinct advantages and limitations. The most conventional method is oral administration, which is non-invasive, convenient, and suitable for long-term treatment, but its therapeutic potential is hindered by poor solubility, extensive first-pass metabolism, and degradation within the gastrointestinal tract [149]. Although strategies such as solid dispersion formulations have shown promise in improving solubility and dissolution rates, their impact on intestinal permeability remains limited, highlighting the persistent challenge of achieving optimal bioavailability for effective post-stroke recovery [156]. Intravenous (IV) administration bypasses the limitations of poor solubility and first-pass metabolism by delivering isorhamnetin directly into the bloodstream, allowing for rapid systemic distribution and immediate bioavailability. Advances in formulation strategies, such as the use of solubilizing agents, have improved its pharmacokinetic properties, allowing for more precise dosing and in vivo investigation. However, limitations remain, including the necessity for medical supervision, the risk of systemic side effects, and the relatively short half-life, which requires frequent dosing [157].

Given these challenges, recent advances in drug delivery have turned to hydrogels as a promising alternative for phytotherapeutic administration. Their biocompatibility and tunable properties allow for targeted delivery at the site of brain injury, minimizing systemic toxicity and enhancing neuroprotection [36]. The exact process of creating hydrogel-based isorhamnetin therapeutic agents has yet to be defined, but delivery would begin with encapsulating the compound within a biodegradable matrix, such as those made from natural polymers like collagen, hyaluronic acid, or synthetic PEG or PVA-based hydrogels [158]. The hydrogel can be administered via intracerebral injection [159], intranasal delivery [160], or as an implantable scaffold [161] near the affected brain region, each with distinct advantages and limitations. Intracerebral injection of hydrogels allows for precise, localized delivery of therapeutic agents directly into the stroke cavity, ensuring high bioavailability and sustained tissue repair while minimizing systemic exposure; however, its invasiveness carries risks of procedural trauma, gliosis modulation, and potential immune activation, necessitating careful hydrogel optimization [162]. In contrast, intranasal delivery offers a non-invasive route that leverages the olfactory and trigeminal pathways to transport therapeutic agents to the brain, reducing procedural risks but facing challenges such as limited drug retention and variability in absorption [163]. Alternatively, implantable hydrogel scaffolds provide a sustained and localized release of therapeutic agents near the affected brain region, supporting tissue regeneration and neuroprotection. However, their placement may still require minor surgical intervention, and their long-term stability and degradation rates must be carefully optimized to match the progression of post-stroke recovery [164]. Once in place, the hydrogel could gradually release isorhamnetin in response to environmental cues such as pH, enzymatic activity, or temperature, ensuring a sustained therapeutic effect that enhances neuronal survival, reduces oxidative stress, and modulates neuroinflammation [39]. Additionally, such hydrogels can be engineered to co-deliver other neuroprotective agents [56], growth factors [165], or stem cells [40], further promoting post-stroke neuroregeneration. One particularly promising approach is the use of injectable thermosensitive hydrogels, which remain in liquid form at room temperature but solidify upon injection into brain tissue, allowing for minimally invasive administration [166]. One example of an injectable thermosensitive hydrogel is poly(N-isopropylacrylamide) (PNIPAAm), often used in biomedical applications due to its thermosensitive behavior. PNIPAAm hydrogels transition from liquid to solid at body temperature due to their lower critical solution temperature (LCST) of around 32 °C [167]. This feature makes them ideal for minimally invasive procedures, as they can be injected in a liquid state at room temperature and then undergo a phase transition to form a gel upon reaching the warmer internal temperatures of the body. This characteristic allows the hydrogel to effectively encapsulate and then gradually release therapeutic agents directly at the target site within the brain, offering sustained delivery that is crucial for long-term treatment and recovery. Due to the limited number of studies examining the full potential of isorhamnetin in stroke therapy, our research team is currently undertaking preclinical studies to evaluate its efficacy using a mouse model of post-stroke depression. The goal is to ascertain whether isorhamnetin can effectively reduce the size of infarcts, mitigate neurological deficits, and improve cognitive and motor recovery outcomes after an ischemic event. By documenting the pharmacokinetics, pharmacodynamics, and potential therapeutic benefits of isorhamnetin in a controlled preclinical setting, we aim to build a foundation for future trials.

## 6. Clinical Translatability

Examining clinical trial registries is essential to gain a comprehensive understanding of the status of research into phytochemical hydrogel-based stroke therapies. A review of the literature on phytochemical hydrogel applications in stroke therapy revealed an extremely limited number of clinical trials on this topic. A PubMed search using the keywords “phytochemicals”, “hydrogel” for studies from the past twenty-five years yielded a total of 30 entries, which include no meta-analysis, 4 reviews, 1 systematic review, and 6 clinical trials, of which 2 are randomized controlled trials. Adding in the keyword “stroke” yielded no results. Upon reviewing the National Institutes of Health’s Clinical Trials.gov registry for phytotherapeutic hydrogel-based stroke therapy studies as of the 5 February 2025, it was notable that there are no ongoing clinical trials in this field. This contrast highlights the significant translational gap between preclinical studies and clinical trials and the need for additional, more standardized preclinical trials of these promising molecules.

The difficulties in transferring promising hydrogel-based and phytotherapeutic hydrogel treatments from the lab to clinical practice are evident. There are still many obstacles to overcome, such as maximizing drug release mechanisms, ensuring biocompatibility, and ensuring production quality, standards, and volume [168]. Additionally, the lack of valid animal models that accurately replicate human stroke pathology, considering factors such as age, comorbidities, and behavioral responses, limits the predictive value of preclinical studies [169]. Variability in study designs, including inconsistent behavioral assessments and diverse outcome measures, further complicates the translation of findings. A concentrated effort is thus required to develop more complex and representative stroke models that account for these factors, thereby more accurately predicting human recovery. Despite these obstacles, the field of hydrogel-based stroke therapy, particularly phytotherapeutic hydrogels, is anticipated to grow as hydrogel technology advances. More creative and effective formulations are probably going to appear as hydrogel technology, and our knowledge of bioactive phytochemicals will develop.

## 7. Current Trends in Hydrogel Therapy for Stroke Recovery

The exploration of hydrogels and phytochemicals in stroke therapy marks a pivotal advancement in the realm of biomedical research, offering a sophisticated approach to overcoming the inherent limitations of traditional treatments for ischemic and hemorrhagic strokes. Traditional therapies such as tissue plasminogen activator and endovascular thrombectomy, despite their effectiveness, are constrained by narrow therapeutic windows and potential risks of complications [58]. Similarly, standard surgical interventions for hemorrhagic stroke, like hematoma evacuation and aneurysm clipping, while crucial, often lead to less-than-optimal recovery outcomes.

Emerging strategies like genetic cell conversion, stem cell therapy, and molecule-loaded hydrogels aim to overcome these traditional limitations. Hydrogels in particular stand out for their targeted drug delivery capabilities, enabling precise localization of therapeutic agents directly at the injury site, thus maximizing therapeutic efficacy while minimizing systemic side effects [37]. These hydrogels, which include sophisticated materials such as polyethylene glycol, alginate or chitosan, are designed to mimic the extracellular matrix, enhancing biocompatibility, drug bioavailability, and sustained release of therapeutic agents [36]. The multifunctional role of hydrogels extends beyond drug delivery; they contribute to neuroprotection, tissue repair, angiogenesis, and synaptic plasticity, and are instrumental in modulating inflammatory responses and stabilizing the blood–brain barrier [170].

Moreover, the integration of phytotherapeutic compounds like curcumin, tannic acid, gallic acid, ginsenosides, resveratrol, and notably, isorhamnetin into hydrogel systems significantly enhances their therapeutic potential. The limited bioavailability and rapid metabolism of these phytochemicals necessitate the use of advanced delivery systems such as nanocarriers and hydrogels to optimize their therapeutic efficacy. For instance, curcumin-loaded hydrogels help regulate microglial polarization, reducing post-stroke neuroinflammation and aiding rehabilitation. Similarly, tannic acid-based hydrogels promote neuroplasticity and motor recovery by modulating microglial responses and reducing oxidative stress, as detailed in research findings [171]. Isorhamnetin, known for its anti-inflammatory, antioxidant, and anti-apoptotic properties, plays a pivotal role in mitigating oxidative stress and inflammation, thereby augmenting the neuroprotective effects that are vital for effective stroke recovery [139]. The ability of isorhamnetin-enriched hydrogels to modulate cellular environments post stroke significantly supports neuroplasticity and neural repair [171]. However, the absence of standardized protocols for dosage, administration methods, and timing of interventions further complicates their clinical application.

Importantly, the translation of hydrogel-based phytochemical treatments from preclinical models to clinical trials faces a number of challenges. The complexity of human pathophysiology, including factors like patient age, comorbidities, and the limitations of murine models, complicates direct applicability [169]. Moreover, while hydrogels have demonstrated efficacy in applications such as skin and bone regeneration, their use in nerve and brain tissue represents a newer frontier [58]. The intricate task of not only restoring the biological and molecular integrity of brain tissue but also its functional capabilities poses an immense challenge. Hydrogels must be designed to not only deliver therapeutic agents effectively but also to ensure that these agents can operate in a systemically and regionally appropriate manner that aligns with the complex healing processes required for neurological recovery [168]. More importantly, the molecular and cellular recovery of the affected area is not sufficient, as the full recovery of function (cognitive/motor) should be the end goal of a successful therapy [172]. Furthermore, several other challenges reduce the clinical translation of hydrogel-based and phytotherapeutic therapies, such as numerous immunogenic risks [173], difficulties in controlling drug release kinetics [174], and scalability issues [175] that remain significant obstacles. The absence of clinical trials specifically investigating phytochemical-loaded hydrogel therapies for the treatment of stroke highlights this translation gap.

Despite the promising preclinical outcomes of hydrogel-based therapies for stroke recovery, several challenges remain in terms of their clinical translation. Critical issues include the need for standardized formulation protocols, reproducible manufacturing processes, and comprehensive long-term safety assessments, especially considering the dynamic post-stroke environment [176]. Additionally, optimizing drug release kinetics and ensuring mechanical stability over extended periods remain significant hurdles. Looking ahead, future research should focus on developing smart, stimulus-responsive hydrogels and integrating advanced nanotechnologies to enable multi-drug delivery [39]. Moreover, hydrogel therapy could morph into a highly sophisticated and highly personalized therapeutic strategy that could in term be dependent on individual disease characteristics. Therefore, creating a facility that could successfully support sophisticated bioprinting together with highly specialized cell culture facilities could prove to be expensive and difficult to sustain. Moreover, this strategy will demand a vast multidisciplinary team—including clinicians, engineers, chemists, and biologists—to overcome regulatory, scalability, and patient-specific treatment optimization challenges, while also addressing issues such as batch-to-batch consistency, potential immunogenicity, and the gap between preclinical models and human pathophysiology. In the long term, the potential for growth within this field could, however, be immense. Formulations developed for the central nervous system could have transformative applications in peripheral damage and other medical conditions. However, the successful expansion and adaptation of these technologies require the formation of highly skilled interdisciplinary teams. Such collaborations are essential to advancing our understanding of pathophysiology and refining the integration of hydrogel formulations with phytochemicals.

## 8. Conclusions

In conclusion, the integration of hydrogels and phytochemicals represents a transformative approach in the treatment of stroke, offering new avenues for overcoming the inherent limitations of conventional therapies. The application of hydrogels provides a sophisticated platform for targeted and sustained delivery of therapeutic agents, which not only maximizes efficacy but also minimizes side effects, thus addressing key challenges associated with traditional stroke treatments. Moreover, the incorporation of phytochemical compounds leverages their neuroprotective properties to further enhance recovery outcomes, supporting neurogenesis and reducing neuroinflammation while addressing limitations such as poor bioavailability and absorption. This combination of advanced biomaterial technologies and natural molecules could pave the way for personalized and effective stroke therapies, improving both the life expectancy and the quality of life of stroke survivors. As the field progresses, continued interdisciplinary research and clinical trials will be crucial in refining these approaches, ensuring their safety, efficacy, and accessibility to fundamentally improve stroke recovery and rehabilitation options.

## Figures and Tables

**Table 1:** Curcumin-based studies.

Name of the Hydrogel	Method of Preparation	Summary of the Research Work (Novelty)	References
Curcumin-loaded hydrogel with ROS scavenging	Double ROS-scavenging hydrogel; likely involves dual-crosslinking mechanisms for sustained release and antioxidant activity.	Regulates microglia activity and enhances stroke rehabilitation through a sustained curcumin release mechanism.	Zhang et al., 2024 [80]
Curcumin-loaded pH-sensitive biopolymer hydrogels	pH-sensitive biopolymer hydrogel, responds to acidic microenvironments; likely ionic crosslinking for controlled release.	Designed to release curcumin in response to pH variations, ensuring targeted delivery in ischemic stroke models.	Yang et al., 2022 [81]
NT-3 and curcumin delivery via spinal cord matrix hydrogel	Decellularized spinal cord matrix hydrogel, mimicking ECM structure; physical crosslinking with embedded growth factors.	Supports neural regeneration and neuroprotection through a biomimetic scaffold incorporating NT-3 and curcumin.	Chen et al., 2024 [83]
Curcumin-encapsulated nanoparticles in injected hydrogels	Chitosan-based injectable hydrogels with PEG–PCL grafting; hydrophobic interactions for nanoparticle retention.	Enhances bioavailability and retention of curcumin nanoparticles within ischemic stroke regions.	Madech et al., 2023 [84]
Supramolecular hydrogel for curcumin and edaravone release in ischemic stroke	Supramolecular hydrogel, non-covalent interactions, providing dynamic, self-healing properties.	Facilitates controlled, long-term release of neuroprotective agents in ischemic stroke therapy.	Jia et al., 2023 [85]

**Table 2:** Tannic acid-based studies.

Name of the Hydrogel	Method of Preparation	Summary of the Research Work (Novelty)	References
Tannic acid as a crosslinker for functional polymeric networks	Tannic acid used as a crosslinking agent; forms hydrogen bonds with polymers, enhancing mechanical strength.	Investigates the role of tannic acid in forming robust polymeric hydrogel networks with improved stability.	Chen et al., 2022 [87]
Tannic acid-enriched eco-friendly hydrogels	Tannic acid-crosslinked hydrogels; biocompatible and biodegradable for biomedical applications.	Develops sustainable, eco-friendly hydrogels for potential biomedical and wound-healing applications.	Kaczmarek et al., 2020 [88]
Collagen/beta-glucan hydrogels crosslinked with tannic acid	Hydrogen bonding and physical crosslinking between tannic acid and collagen, enhancing mechanical properties.	Enhances the bioactivity and structural integrity of collagen-based hydrogels for tissue engineering.	Michalska-Sionkowska et al., 2021 [89]
Wearable tissue adhesive hydrogel with tannic acid	Ternary hydrogel composed of tannic acid, chitosan, and polyacrylamide; provides adhesive and flexible properties.	Designed for wearable biomedical applications with strong adhesion and tissue compatibility.	Yu et al., 2022 [91]
pH-responsive tannic acid–agarose composite hydrogels	pH-responsive hydrogel; ionic crosslinking enhances antibacterial and anti-inflammatory effects.	Aims to develop responsive hydrogels for controlled drug delivery and antimicrobial applications.	Ninan et al., 2016 [92]
Tannic acid-based injectable hydrogel regulating microglial phenotype	Injectable hydrogel with nanoarchitectonic design; promotes neuroplasticity for post-stroke rehabilitation.	Investigates the neuroprotective effects of tannic acid-based injectable hydrogels in stroke recovery.	Liu et al., 2023 [95]

**Table 3:** Gallic acid-based studies.

Name of the Hydrogel	Method of Preparation	Summary of the Research Work (Novelty)	References
Gallic acid-loaded chitosan nanoparticles for ischemic stroke	Chitosan nanoparticle hydrogel; promotes sustained release; electrostatic interactions for improved bioavailability.	Investigates the neuroprotective effects of gallic acid in a chitosan-based hydrogel system designed for ischemic stroke therapy.	Zhao et al., 2020 [104]
Pyrogallol-containing hydrogel with gallic acid	Pyrogallol-functionalized hydrogel; likely uses covalent bonding for structural integrity.	Enhances antioxidant activity and structural stability for biomedical applications.	Weian et al., 2024 [105]
Poly (citrate–gallic acid)–exosome hybrid hydrogel	Hybrid hydrogel combining polymer–exosome structures for enhanced neuro-restoration.	Develops a regenerative hydrogel system combining gallic acid derivatives with exosomes to improve neuroprotection and functional recovery.	Li et al., 2022 [106]

**Table 4:** Ginsenoside-based studies.

Name of the Hydrogel	Method of Preparation	Summary of the Research Work (Novelty)	References
Ginsenoside Rg1 nanoparticles activating angiogenesis	Nanoparticle-loaded hydrogel promoting epigenetic modifications and angiogenesis.	Investigates the role of Ginsenoside Rg1 in enhancing neurovascular remodeling and angiogenesis post-ischemic stroke.	Shang et al., 2022 [116]
Ginsenoside Rg3 in hyaluronic acid thermosensitive hydrogel	Thermosensitive hydrogel based on Poloxamer 407 and hyaluronic acid for skin wound healing.	Utilizes a biocompatible hydrogel for controlled release of Ginsenoside Rg3, accelerating wound-healing processes.	Peng et al., 2022 [125]
Ginsenoside Rg3 nanoparticles in thermosensitive hydrogel for cancer therapy	Chitosan derivative-based hydrogel with thermosensitive properties for localized drug delivery.	Develops a targeted drug delivery system for cancer therapy, ensuring sustained release of Ginsenoside Rg3 at tumor sites.	Wu et al., 2022 [127]

**Table 5:** Resveratrol-based studies.

Name of the Hydrogel	Method of Preparation	Summary of the Research Work (Novelty)	References
Resveratrol nanostructured hydrogel for brain targeting	In situ nanostructured hydrogel, promotes brain-targeted delivery via nanoparticle encapsulation.	Enhances bioavailability and targeted delivery of resveratrol to the brain for neuroprotection and therapeutic effects.	Rajput et al., 2018 [135]
Hyaluronic acid hydrogel with resveratrol-loaded chitosan nanoparticles	Hyaluronic acid hydrogel for skin applications, combined with chitosan nanoparticles for enhanced permeability.	Develops a skin-friendly hydrogel system incorporating resveratrol for improved dermal penetration and antioxidant effects.	Conte et al., 2023 [136]
Composite hydrogel with resveratrol-laden nanoparticles for wound healing	Composite hydrogel integrating platelet-derived extracellular vesicles for regenerative purposes.	Combines bioactive extracellular vesicles with resveratrol for enhanced tissue regeneration and wound healing.	Zhu et al., 2022 [137]

**Table 6:** Isorhamnetin-based studies.

Name of the Hydrogel	Method of Preparation	Summary of the Research Work (Novelty)	References
Hydrogel-modified chromatography for isolating active ingredients	Hydrogel-modified chromatography system, facilitates selective purification of bioactive compounds.	Enhances the efficiency of active ingredient isolation by integrating hydrogel-based modifications in chromatographic techniques.	Huang et al., 2023 [150]
Isorhamnetin glycoside (IRGs)-loaded gelatin (GL) nanofiber mats	Fabrication of gelatin nanofibers loaded with Opuntia ficus-indica flour (12% *w*/*v*) by forcespinning, using 25% and 30% (*w*/*v*) GL solutions, followed by glutaraldehyde crosslinking.	Development and characterization of IRG-loaded gelatin nanofibers with controlled release and cytocompatibility, providing a novel delivery system for phytochemicals with potential biomedical applications.	García-Valderrama et al., 2022 [153]
Isorhamnetin-loaded biogenic silica (frustules) from *Cyclotella* sp.	Encapsulation of isorhamnetin into diatom-derived silica frustules using a novel microfluidic device fabricated by ESCARGOT technology, optimizing drug concentration and residence time.	Development of a reproducible microfluidic-based method for loading hydrophobic drugs into biogenic silica carriers, demonstrating controlled isorhamnetin release and potential for standardized drug delivery systems.	Marcia-Andrade et al., 2019 [154]

## Data Availability

Not applicable.
